# Translating and establishing the psychometric properties of the Jenkins Sleep Scale for Arabic-speaking individuals

**DOI:** 10.1186/s12888-024-05714-2

**Published:** 2024-03-28

**Authors:** Yasmin AlMashouk, Salma Yasser Abu-Saleh, Hadeel Ghazzawi, Khaled Trabelsi, Haitham Jahrami

**Affiliations:** 1 Independent researcher, Manama, Bahrain; 2 Department Nutrition and Food Technology, Jordan University of Science and Technology, Irbid, Jordan; 3 Nutrition and Food Science Department, Agriculture School, The University of Jordan, Amman, P. O. Box 11942, Jordan; 4 High Institute of Sport and Physical Education of Sfax, University of Sfax, 3000 Sfax, Tunisia; 5 Research Laboratory: Education, Motricity, Sport and Health, EM2S, LR19JS01, University of Sfax, 3000 Sfax, Tunisia; 6 Government Hospitals, Manama, Bahrain; 7 Department of Psychiatry, College of Medicine and Medical Sciences, Arabian Gulf University, Manama, Bahrain

**Keywords:** Arabic, Jenkins Sleep Scale, Psychometrics, Translation, Validation

## Abstract

**Background:**

The Jenkins Sleep Scale is a widely used self-report questionnaire that assesses sleep quality and disturbances. This study aimed to translate the scale into Arabic and evaluate its psychometric properties in an Arabic-speaking population.

**Methods:**

The Jenkins Sleep Scale was translated into Arabic using forward and backward translation procedures. The Arabic version was administered to a convenience sample of 420 adults along with the Pittsburgh Sleep Quality Index (PSQI) and Athens Insomnia Scale (AIS) for validation purposes. Reliability was examined using Cronbach’s alpha and McDonald’s omega coefficients. Confirmatory factor analysis (CFA) was also conducted to test the unidimensional factor structure. Convergent validity was assessed using correlations with PSQI and AIS scores.

**Results:**

The Cronbach’s alpha and McDonald’s omega values for the Arabic Jenkins Sleep Scale were 0.74 and 0.75, respectively, indicating good internal consistency. The 2-week and 4-week test-retest intraclass correlation coefficients were both 0.94 (*p* < 0.001), indicating excellent test-retest reliability. The CFA results confirmed the unidimensional factor structure (CFI = 0.99, TLI = 0.96, RMSEA = 0.08). The measurement model had an equivalent factor structure, loadings, intercepts, and residuals across sex, age, and marital status. Significant positive correlations were found between the Arabic Jenkins scale score and the PSQI (*r* = 0.80, *p* < 0.001) and AIS (*r* = 0.74, *p* < 0.001), supporting convergent validity.

**Conclusion:**

The Arabic version of the Jenkins Sleep Scale demonstrated good psychometric properties. The findings support its use as a valid and reliable measure for evaluating sleep quality and disturbances among Arabic-speaking populations.

**Supplementary Information:**

The online version contains supplementary material available at 10.1186/s12888-024-05714-2.

## Introduction

Sleep is a vital component of health and well-being. Insufficient sleep can negatively impact physical health, mental health, overall functioning, and quality of life [1]. It is estimated that almost one-third of the general population suffers from insomnia symptoms [2, 3], and between 8 and 18% of people are dissatisfied with their sleep quality or quantity [2]. Recent global events have necessitated an emphasis on sleep in practice and research, as approximately 40% of the general population and 75% of COVID-19 patients experienced sleep problems during the pandemic [4]. The persistence of sleep disturbances after recovery has also been observed [5].

High rates of sleep deprivation are particularly prevalent across the Arab world [6–10]. This could be attributed to much of the population being unaware of the serious health implications of disordered sleep [11]. Within the region, poor sleep quality and quantity have been implicated as risk factors for obesity [12–14], as well as road traffic collisions [15, 16]. Moreover, emerging patterns of problematic sleep habits in adolescents have been found in the United Arab Emirates [17], Saudi Arabia [18, 19] and Oman [20]. Evidently, sleep disturbances are pervasive, yet a lack of knowledge regarding sleep medicine is common among healthcare professionals in some Arab countries [11]. Primary care physicians have also been shown to underrecognize the importance of sleep disturbances, creating barriers to treatment [21–24].

Sleep is often neglected in primary care settings even outside of Arabic-speaking countries [25]. To remedy this, Senthilvel et al. (2011) suggested the use of validated sleep questionnaires prior to consultation as a quick and effective method to screen patients for sleep disturbances [26]. Sleep research is underdeveloped in the Arab world [27]; therefore, having access to psychometrically sound measures for assessing sleep is important in both research and clinical contexts.

The Jenkins Sleep Scale (JSS) is a commonly used self-report questionnaire that evaluates sleep difficulties and disturbances and is not limited to a specific clinical group [28]. It consists of four items that assess trouble falling asleep, trouble staying asleep, waking up several times per night, and waking up feeling tired and worn out. Participants rated each item on a 6-point scale ranging from 0 (never) to 5 (every day) to indicate how often they experienced each sleep problem over the past month. Higher total scores indicate poorer sleep quality and increased sleep disturbances. The JSS has demonstrated strong reliability and validity across various populations and has been translated into a number of languages [28–33]. Specifically, the Cronbach’s alpha values for the JSS translated and validated across languages, including English [28], Portuguese [29], Urdu [31], Turkish [30, 34, 35], Spanish [33, 36], German [37], and Finnish [32], ranged from 0.63 to 0.90. The lowest alpha was detected in the English version tested in patients (0.63) from the 1988 study by Jenkins [28], while the highest alpha was detected in the German version tested in healthy subjects (0.90) from the 2020 study by Tibubos [37]. The JSS showed good internal consistency across different language versions and populations, including both healthy individuals and patients, indicating that the JSS is a reliable instrument for assessing sleep disturbances. The Cronbach’s alpha meets the recommended criterion of > 0.70 in the majority of studies, supporting the use of the JSS as a psychometrically sound measure across cultures [28–33].

The JSS has yet to be translated and validated for use among Arabic-speaking populations. The purpose of this study was to translate the JSS into Arabic and evaluate the psychometric properties of the translated version among Arabic speakers to provide a tool that Arabic-speaking clinicians and researchers can readily use. To address this gap, the current study aimed to translate the JSS into Arabic and assess its psychometric properties among Arabic-speaking populations.

The number of validated sleep assessment scales in Arabic is scarce. This represents a significant research gap, as the availability of psychometrically robust tools in Arabic is crucial for accurately evaluating sleep issues in Arabic-speaking populations. In light of this gap, our study aimed to address this need by conducting the first validation of the Arabic version of the JSS. By undertaking this research, we aim to contribute to the advancement of sleep assessment practices in Arabic-speaking populations and improve the accuracy of sleep evaluations in these contexts.

The brief nature of the JSS makes it a highly suitable tool for rapid use, especially during challenging times such as pandemics or natural disasters. Its efficiency allows for efficient data collection and quick assessments of sleep quality, making it a valuable resource in situations where timely evaluation is critical.

## Methods

### Participants and data collection

A convenience sample of 420 participants living in a general community setting was obtained through recruitment on various social media platforms, such as [Discord, Facebook, Instagram, LinkedIn, Pinterest, and Twitter/X], as well as instant messaging services, including [LINE, Telegram, Viber, and WhatsApp]. The inclusion criteria were age 18 years or older and the ability to read and comprehend Arabic. Participants completed an online questionnaire via Google Forms, which included basic demographic questions (age, sex, height, weight, and marital status), the Arabic JSS, the Arabic version of the Pittsburgh Sleep Quality Index (PSQI) [38], and the Arabic version of the Athens Insomnia Scale (AIS) [39]. A subsample of the participants (*n* = 147) completed the questionnaire again at two- and four-week intervals for test-retest reliability. To ensure the test-retest reliability of our measures, participants were requested to provide their email addresses during the initial survey administration. The inclusion of email addresses allowed us to recontact participants for the retest portion of the study, facilitating the assessment of the stability and consistency of the measures over time. To minimize the unnecessary burden on the entire sample, we made a deliberate decision to include only one third of the original sample (35% of 420 participants) for the test-retest reliability assessment. To select participants for the retest validity assessment, we employed a simple random sampling technique. Specifically, we utilized a random starting point and selected every third participant from the initial sample. This sampling approach was chosen to ensure the representation of a diverse and unbiased subset of participants in the retest phase. The email addresses provided by participants served as a crucial tool in matching the initial test responses with the corresponding retest responses, enabling us to establish the test-retest reliability of the measures. Furthermore, this matching process played a pivotal role in preventing the inclusion of duplicate data, ensuring the integrity, consistency, and accuracy of the obtained results. The PSQI [40] and AIS [41, 42] were included to assess convergent validity. The PSQI is a questionnaire that measures sleep quality and disturbances over a one-month period [40]. It consists of 19 self-report questions and five questions rated by the participant’s bed partner or roommate [40]. These items constitute seven components that are routinely examined in clinical sleep assessments, including subjective sleep quality, sleep latency, sleep duration, habitual sleep efficiency, sleep disturbances, use of sleeping medications, and daytime dysfunction [40]. Total scores range from 0 to 21, with higher scores indicating worse sleep quality [40]. The Arabic version of the PSQI has shown acceptable internal consistency (⍺ = 0.65). The AIS is an 8-item questionnaire that assesses sleep within the past month according to the International Classification of Disease-10 (ICD-10) criteria for insomnia [42]. Total scores ranged from 0 (absence of any sleep-related problem) to 24 (a severe degree of insomnia) [43]. The scale has also demonstrated reliability as a screening tool for insomnia [41], and the Arabic version has shown good psychometric properties (⍺ = 0.83) [38].

### Sample size calculation and power analysis

A sample size of approximately 100 was deemed sufficient to conduct factor analysis on the 4 items of the JSS. Factor analysis is used to identify underlying factors or dimensions between measured variables. Recommendations for minimum sample sizes vary but generally suggest having at least 5–10 observations per item and an overall sample size of at least 100–200. With 4 items on the JSS, a sample size of approximately 100 meets these recommendations. Having at least 100 respondents provides a sufficient number of observations to allow for reliable statistical analysis and detection of major underlying factors among the JSS items.

To avoid issues with missing data, all questions in the survey were required to be answered by participants to complete the questionnaire. This mandatory response format ensured complete data for all respondents. No monetary or nonmonetary incentives were offered to the participants for their involvement in the survey.

### Ethical issues

This study was approved by the Government Hospitals, Bahrain Institutional Review Board Code (2023/1478), and all participants provided informed consent prior to participation. Informed consent was obtained from all subjects through an electronic process. Participants were required to provide their informed consent by checking a box to indicate their agreement with the study’s terms and conditions. The study procedures adhered to the ethical guidelines outlined in the Helsinki Declaration of 1964 and its subsequent amendments (1975, 1983, 1989, and 1996). Informed consent was obtained from all subjects. The data were reported only in aggregate form. Participants were informed of the data used, confidentiality protocols, and security measures taken to prevent unauthorized access to the survey responses.

### The translation process

The English version of the JSS was translated into Arabic following established forward-back translation methods to ensure accuracy and cultural relevance [44]. Two bilingual translators independently translated the scale from English into Arabic. The two Arabic versions were subsequently compared by a third independent bilingual translator to resolve inconsistencies and synthesize one translated version, with consensus among the two previous translators and the research team. Following this, a blind back-translation was performed in which two new bilingual translators, who were completely blind to the original version of the JSS, independently translated the Arabic version back into English. Discrepancies between the back-translated versions and the original English version were examined and resolved by an expert multidisciplinary committee. All members involved in the forward-backward translation process held a doctoral degree and had extensive experience conducting sleep medicine research, ensuring high-quality translation that preserved the nuances and technical meanings of the original scale.

To confirm conceptual and semantic consistency, Cohen’s kappa coefficient [45] was computed to assess the level of agreement between the translators for each item on the questionnaire. This value was found to be > 0.99, indicating almost perfect agreement [46]. A pilot study was conducted involving 30 Arabic-speaking participants who were acquaintances or family members of the research team. No changes were made to the questionnaire during the pilot study. Feedback from all 30 participants indicated that the translated survey was well understood and comprehensible.

### Statistical analyses

Preliminary analyses included descriptive statistics (mean, standard deviation, skewness, and kurtosis) for all the measures. Skewness and kurtosis were used to determine the normality of the data, with values between − 2 and + 2 being set as cutoff points [47]. Values within this range are considered acceptable levels of skewness and kurtosis for assuming a normal distribution. Sex, age, and marital status differences on the Arabic JSS were assessed using independent sample t tests. Age was divided into two groups, those younger than 35 years and those older than 35 years, based on the median age.

The internal consistency of the Arabic JSS was evaluated using Cronbach’s alpha [48] and McDonald’s omega [49] coefficients. Values of 0.70 or higher were considered satisfactory [50]. To evaluate the test-retest reliability of the JSS, we calculated intraclass correlation coefficients (ICCs) between JSS scores at two time points (i.e., test vs. retest at 2 weeks AND test vs. retest at 4 weeks). To assess construct validity, confirmatory factor analysis (CFA) using the maximum likelihood extraction technique [51] was conducted to test the unidimensional factor structure of the Arabic JSS. Model fit was assessed using the comparative fit index (CFI), Tucker‒Lewis index (TLI), and root mean square error of approximation (RMSEA). CFI and TLI values above 0.90 and an RMSEA less than 0.08 indicated acceptable model fit [52]. To evaluate the generalizability and comparability of our findings, we conducted a series of measurement invariance tests using multigroup CFA across sex, age, and marital status. Configural, metric, scalar, and residual (strict) invariance were examined by systematically imposing equality constraints and evaluating changes in fit indices used in global CFA (i.e., CFI, TLI, RMSEA, and SRMS) [53]. A CFI difference (ΔCFI) of less than 0.01 and an RMSEA difference (ΔRMSEA) of less than 0.015 indicated no significant decrease in fit between models [53]. A significant chi-square difference test indicated that invariance could not be assumed [53]. For configural invariance, we specified the same CFA model across groups with no equality constraints imposed [53]. This model had an adequate fit with conventional criteria (CFI > 0.90, RMSEA < 0.08) [53]. Metric invariance was supported if ΔCFI and ΔRMSEA were less than the cutoff, and the chi-square difference test was nonsignificant after constraining factor loadings to be equal [53]. Scalar invariance was supported after additionally constraining intercepts equal, with ΔCFI < 0.01, ΔRMSEA < 0.015, and a nonsignificant chi-square difference test [53]. Residual invariance was supported after constraining residual variances equal, with minimal changes in fit [53].

To examine convergent validity, Pearson’s correlation coefficients were calculated between total scores on the Arabic JSS and total scores on the PSQI and AIS. Strong positive correlations were expected based on these instruments’ assessments of similar sleep constructs.

A network analysis was conducted on the data from the 4-item JSS questionnaire. The JSS includes questions on trouble falling asleep (JSS-1), waking up during the night (JSS-2), having trouble staying asleep (JSS-3), and not getting enough rest from sleep (JSS-4) [54]. The network consisted of 4 nodes, one for each JSS item. Edges were defined between nodes based on Pearson correlation coefficients between all pairs of JSS items, with edges retained between nodes with correlations greater than 0.3 [54].

Several common network analysis metrics were calculated to characterize the centrality and interconnectedness of nodes. The nodal centrality measures included betweenness centrality, closeness centrality, node strength, expected influence, Barrat’s measure, Onnela’s measure, weighted symmetrical uncertainty (WSa), and Zhang’s centrality [54]. A high betweenness centrality indicates that the node lies on many of the shortest paths between other nodes, while a high closeness centrality means that the node can reach others quickly [54]. The node strength sums the edge weights connected to the node. The expected influence measures the total strength of a node’s neighbors. Barrat’s measure of the node’s weighted degree [54]. The Onnela measure incorporates the intensity and number of links, while the Zhang measure considers indirect as well as direct links [54]. Clustering coefficients were also calculated per node to quantify the interconnectedness between a node and its neighbors [54].

Constructing a network model enables the visualization and quantification of complex associations within the scale’s structure. This provides additional insight compared to traditional techniques such as factor analysis, which focus solely on relationships between items and latent variables. Specifically, network analysis can identify highly interconnected core items, detect clustering, and, through analysis of connections between items, highlight any redundancies or weak associations. Ultimately, network analysis complements conventional psychometric assessments by generating a more nuanced understanding of the underlying relationships and connections between scale items themselves.

Based on guidelines for network analysis in psychometric studies, we applied an edge weight cutoff of 0.25 [55, 56]. This means that only connections with a partial correlation greater than 0.25 were depicted as edges in the network graph [56]. Applying this cutoff filters out weaker connections and provides a more parsimonious visualization focused on the most relevant associations between items [56]. The specific cutoff value was selected based on prior recommendations for network modeling of psychometric scales to balance detail with interpretability [56].

All analyses were performed using the lavaan package (version 0.6–17) in R (version 4.3.2 (eye holes)) and were released on 2023-10-31. Network metrics were computed using R statistical software and the qgraph package (version 1.9.8). Visualization of the network structure was performed with the same software. The qgraph implements graphical modeling techniques to estimate network connections between items and generates graphical displays of these connections [55]. After estimating a network model, we used qgraph to visualize nodes (scale items) and edges (partial correlation coefficients between items) [55]. The Fruchterman-Reingold algorithm was applied to determine node placement, with strongly connected nodes placed closer together [55]. Network graphs were rendered with nodes color-coded by subscale membership and weighted edges representing the strength of association between items [55]. This approach allowed clear visualization of the overall network structure, clusters of related items, and central nodes [55].

## Results

Of the 420 participants, 71 (17%) were male and 349 (84%) were female. The mean age was 22.66 years (SD = 6.85), and the mean BMI was 23.17 kg/cm^2^ (SD = 4.55). The majority of participants were also single (85%). Further demographic characteristics, as well as the mean scores for each scale, can be found in Table 1. The JSS contains four items, all of which presented acceptable skewness. The average total score for the JSS was M = 5.39 (SD = 3.9). Females (M = 5.41, SD = 4.01) scored higher on the JSS than males did; however, no significant difference was found between their means (*p* = 0.801). Participants younger than 35 years (M = 6.54, SD = 4.01) scored higher than did those 35 years and above, but again, no significant difference was found (*p* = 0.659). Finally, no significant difference was found between the mean scores of married and single participants (*p* = 0.497).

**Table 1:** Descriptive results of the Jenkins Sleep Scale (JSS) (*n* = 420)

Variable	Mean	SD	Skewness	Kurtosis
Age	22.66	6.85	2.85	8.82
Ht (cm)	162.63	9.02	0.52	1.19
Wt (kg)	61.49	14.29	1.23	3.04
BMI kg/cm^2^	23.17	4.55	1.01	1.58
JSS-1	1.19	1.21	1.04	0.7
JSS-2	1.39	1.31	1.05	0.63
JSS-3	1.11	1.2	1.28	1.49
JSS-4	1.7	1.46	0.68	-0.34
JSS Total	5.39	3.9	0.97	1.25
AIS	6.52	4.05	0.56	0.00
PSQI	7.01	3.33	0.12	-0.45

Notes: Results are expressed as arithmetic means and standard deviations. Jenkins Sleep Scale (JSS), Athens Insomnia Scale (AIS) and Pittsburgh Sleep Quality Index (PSQI) were used

The JSS demonstrated good internal consistency, with Cronbach’s alpha and McDonald’s omega values of 0.74 and 0.75, respectively. The intraclass correlation coefficient (ICC) was 0.94, indicating that the test-retest reliability was excellent. See Table 2.

**Table 2:** Reliability analysis coefficients of the Jenkins Sleep Scale (JSS) (*n* = 420)

Variable	Item-rest correlation	Cronbach’s α	McDonald’s ω	Intraclass correlation ICC
Total JSS	NA	0.74	0.75	0.94
If item dropped				
JSS-1	0.56	0.67	0.69	NA
JSS-2	0.44	0.73	0.74	NA
JSS-3	0.64	0.63	0.64	NA
JSS-4	0.52	0.70	0.72	NA

Notes: The ICCs were based on a subsample of 147 participants only

The CFA showed that all four items had factor loadings above 0.40 and were therefore considered stable. These results can be found in Table 3. The model obtained a good fit (X^2^ = 7.66, *p* = 0.022), with fit indices of 0.99 for CFI, 0.96 for TLI, and 0.08 (95% CI: 0.03–0.15) for RMSEA.

**Table 3:** Confirmatory factor analysis of the Jenkins Sleep Scale (JSS) score (*n* = 420)

Factor	Indicator	Estimate (Loading)	SE	Z	*p*
JSS	JSS-1	0.83	0.06	13.59	< 0.001
	JSS-2	0.69	0.07	10.29	< 0.001
	JSS-3	0.95	0.06	15.78	< 0.001
	JSS-4	0.89	0.08	11.83	< 0.001

Notes: The estimate represents factor loading using maximum likelihood extraction (MLE). Χ²(df), *p* = 385.59 (6), *p* = 0.02. The comparative fit index (CFI) = 0.99. The Tucker‒Lewis index (TLI) was 0.96. The Bentler–Bonett Nonnormed Fit Index (NNFI) = 0.96. The Bentler–Bonett normed fit index (NFI) was 0.98. The parsimony-normed fit index (PNFI) was 0.33. The pollen relative fit index (RFI) was 0.94. The increase in the Bollen Fit Index (IFI) was 0.99. The relative noncentrality index (RNI) = 0.99. Log-likelihood = -2624.24. Number of free parameters = 12. Akaike (AIC) = 5272.47. Bayesian (BIC) = 5320.95. Sample-size adjusted Bayesian (SSABIC) = 5282.87. Root mean square error of approximation (RMSEA) = 0.08. RMSEA 90% CI lower bound = 0.03. RMSEA 90% CI upper bound = 0.15. RMSEA p value = 0.14. The standardized root mean square residual (SRMR) was 0.02. Hoelter’s critical *N* (α = 0.05) = 329.5. Hoelter’s critical N (α = 0.01) = 505.98. Goodness-of-fit index (GFI) = 1. The McDonald fit index (MFI) was 0.99. The expected cross-validation index (ECVI) was 0.08. Kaiser–Meyer–Olkin (KMO) test = 0.79. Bartlett’s test of sphericity Χ²(df), *p* = 382.69 (6), *p* < 0.001

The psychometric results supported full measurement invariance across all groups. The configural model demonstrated adequate fit per CFI (> 0.90) and RMSEA (< 0.08) values. Constraining factor loadings, intercepts, and residual variances to equality across groups resulted in minimal, nonsignificant changes in CFI, RMSEA, and chi-square difference tests compared to the configural baseline model. This provides evidence that the measurement model had an equivalent factor structure, loadings, intercepts, and residuals across sex, age, and marital status. The results of the measurement invariance across age groups, sexes, and marital status are shown in Supplementary Material 1.

Convergent validity was assessed by evaluating correlations between the Arabic JSS and AIS scores and between the Arabic JSS and PSQI scores. We found a strong positive correlation between participants’ total JSS score and total AIS score (*r* = 0.74, *p* < 0.001). A strong positive correlation was also found with the total PSQI score (*r* = 0.80, *p* < 0.001). Further intercorrelations can be found in Table 4. These findings provide evidence for the convergent validity of the Arabic JSS, as higher scores were associated with higher scores on established measures of poor sleep quality and insomnia symptoms.

**Table 4:** Intercorrelations of the items on the Jenkins Sleep Scale (JSS) and convergent validity of the Athens Insomnia Scale (AIS) and Pittsburgh Sleep Quality Index (PSQI) (*n* = 420)

	JSS-1	JSS-2	JSS-3	JSS-4	JSS Total	AIS	PSQI
JSS-1	—						
JSS-2	0.31**	—					
JSS-3	0.54**	0.45**	—				
JSS-4	0.46**	0.31**	0.46**	—			
JSS Total	0.75**	0.69**	0.80**	0.76**	—		
AIS	0.55**	0.48**	0.60**	0.59**	0.74**	—	
PSQI	0.64**	0.54**	0.62**	0.61**	0.80**	0.59**	—

Notes: * and ** represent p values < 0.05 and < 0.001, respectively

**Fig. 1:**
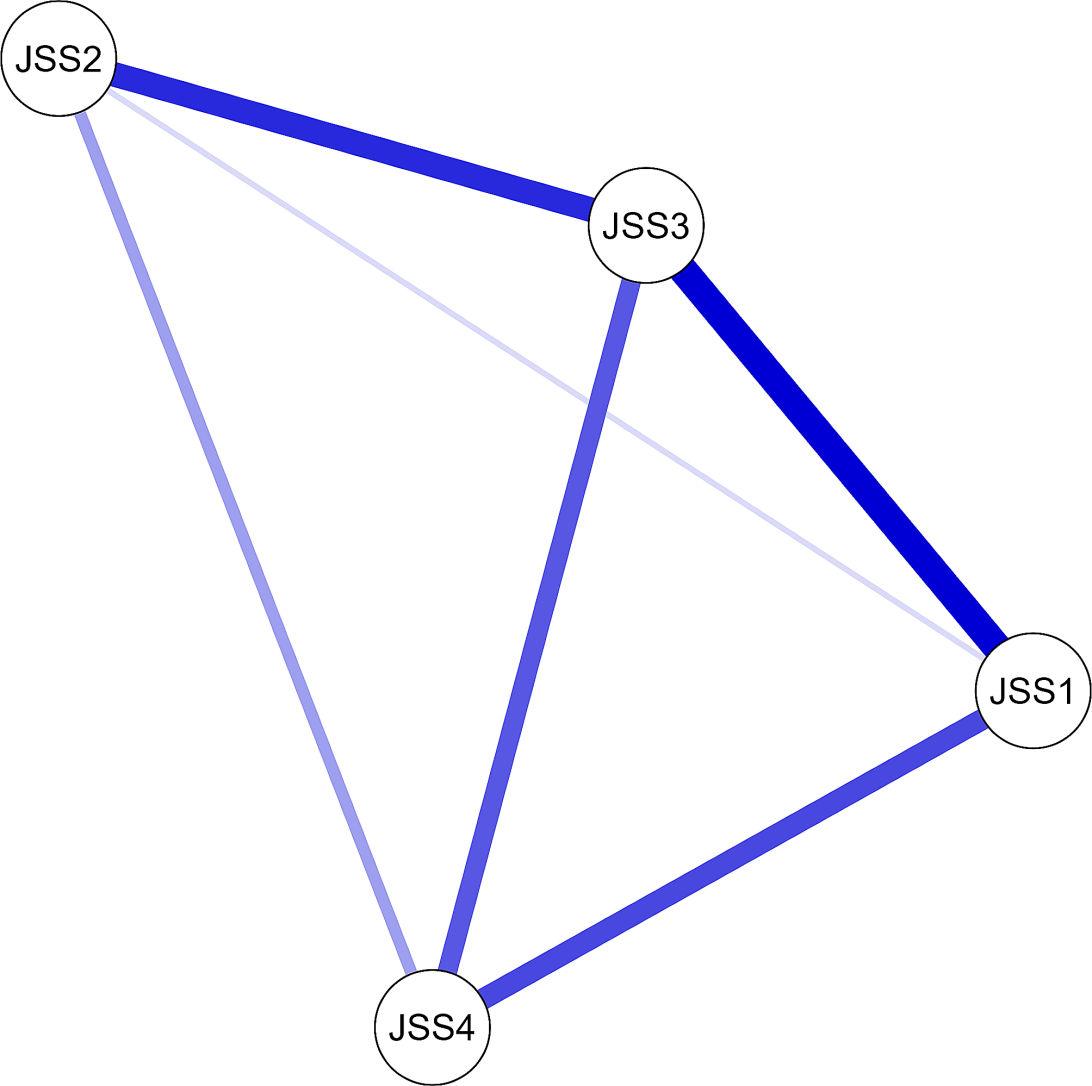
Network analysis of the Jenkins Sleep Scale (JSS) score

The JSS network consisted of 4 densely interconnected nodes. The centrality and clustering measures for each node are shown in the Table below. JSS3 (trouble staying asleep) had the highest betweenness centrality at 1.338, highest closeness centrality at 1.338, and highest overall node strength. See Table 5. These findings indicate that JSS3 is positioned centrally in the network and has a large number of strong interconnections. JSS-1 (trouble falling asleep) had the highest clustering coefficient at 0.214, indicating that its first neighbors are highly interconnected. JSS-4 (lack of restful sleep) had the lowest Barrat score (-1.456), JSS-1 had the highest Onnela score (0.587), and the highest WSa score (0.715). The visual network depiction showed that JSS-1 and JSS3 had the most and strongest linkages to other nodes, respectively. See Fig. 1.

**Table 5:** Network analysis of the Jenkins Sleep Scale (JSS) score (*n* = 420)

Variable	Centrality measures per variable				Clustering measures per variable			
	Betweenness	Closeness	Strength	Expected influence	Barrat^a^	Onnela	WS^a^	Zhang
JSS-1	-0.5	0.214	-0.018	-0.018	0	0.31	0	0.587
JSS-2	-0.5	-0.912	-1.072	-1.072	0	-0.23	0	0.154
JSS-3	1.5	1.318	1.338	1.338	0	-1.234	0	0.715
JSS-4	-0.5	-0.62	-0.247	-0.247	0	1.153	0	-1.456

The coefficient could not be standardized because the variance was too small

## Discussion

This study translated the Jenkins Sleep Scale into Arabic and provided an initial psychometric evaluation of the translated version among a sample of Arabic-speaking adults. The results demonstrated that the Arabic JSS has good reliability and construct validity. The Cronbach’s alpha and McDonald’s omega values were 0.74 and 0.75, respectively, both of which exceeded the 0.70 minimum standard for internal consistency of health measurement instruments [50]. The JSS demonstrated good internal consistency, with a Cronbach’s alpha of 0.74 and McDonald’s omega of 0.75. A value exceeding 0.70 indicates that the JSS items reliably measure the same underlying sleep quality construct. A high ICC of 0.94 for test-retest reliability signifies excellent temporal stability. A high ICC indicates that participants’ JSS scores were consistent over time between the two assessment points. Thus, the internal consistency values support the homogeneity of the scale, while the high test-retest ICCs demonstrate that the JSS provides stable, reproducible scores over time. These metrics of internal consistency and test-retest reliability provide evidence that the JSS has sound psychometric properties and reliably measures self-reported sleep disturbances.

Additionally, the CFA results verified the single-factor structure of the scale, which was consistent with the original version, with fit indices (CFI, TLI, and RMSEA) satisfying their respective benchmarks. These findings suggested that the Arabic JSS effectively measures a clearly defined construct.

Significant positive correlations between the Arabic JSS, PSQI, and AIS provide evidence for convergent validity. The Arabic JSS was related, as expected, to other measures designed to assess similar sleep constructs. These findings suggested that the Arabic JSS accurately captures self-perceived sleep quality and disturbances. The findings indicate that the translated scale is a psychometrically sound tool for evaluating self-reported sleep difficulties among Arabic speakers.

The use of a well-validated sleep assessment tool in Arabic represents a major advance in identifying sleep disturbances among Arabic-speaking populations [57]. Research indicates that sleep disturbances are significantly underrecognized in clinical practice, hampering early diagnosis and treatment [58]. The availability of the Arabic JSS provides clinicians with an evidence-based means of incorporating routine sleep quality evaluation as part of their patient assessments. Early screening facilitates referral to sleep specialists when warranted, enabling access to gold-standard diagnostic testing and treatment [57].

Sleep deprivation is linked to decreased productivity [59] and academic performance [60], which are pronounced in regions where youth make up a significant proportion of the population [4, 10, 11, 27, 43, 61]. A lack of quality sleep can also negatively impact the cognitive skills essential for learning and memory consolidation [62]. Moreover, the strong stigmatization surrounding mental illness in Arab culture makes it critical to recognize links between sleep disturbances and conditions such as anxiety, depression, and suicide risk [63]. Unaddressed sleep disturbances can exacerbate mental health issues [64]. Specific genetic and environmental factors, such as high consanguinity rates and the effects of extreme heat, may further heighten Arab populations’ vulnerability to complications of sleep loss [10, 11, 15, 27]. Thus, uncovering, diagnosing, and treating sleep problems early on is vital to mitigate widespread repercussions on the health, cognitive capacity, productivity, and psychological wellbeing of Arab communities.

The high clustering coefficient for JSS-1 (trouble falling asleep) indicates that this node forms a dense interconnected triad with JSS-3 and JSS-4. Symptoms of sleep-onset insomnia cooccur with trouble staying asleep and feeling unrested. This cluster suggested a pattern of broad sleep impairment.

JSS-2 (awakening during the night) had the lowest clustering coefficient and was the most peripheral node. This finding indicates that mid-sleep awakenings may arise independently of other sleep continuity issues. However, moderate centrality measures that signify night awakenings are somewhat correlated with onset, maintenance, and restorative sleep problems.

Further research on sleep in Arabic-speaking populations is necessary, as disordered sleep is a global public health epidemic [65] for which the Arab world appears to be unprepared [11]. Despite the high rates of sleep deprivation found across the region [6–10], sleep disturbances remain underdiagnosed and overlooked in medicine [11]. Moreover, due to the bidirectional pathway that exists between sleep and psychiatric disorders, neglecting sleep disturbances could impact treatment outcomes and increase the risk of developing a psychiatric disorder [66]. Considering the additional health risks that sleep disturbances pose [12–14], there is a pressing need to address sleep more readily in clinical practice and research. As such, the availability of a concise and easily scored scale, such as the Arabic JSS, has important implications for improving screening practices within primary care and monitoring sleep within clinical populations and allowing researchers to better understand cultural variations within disordered sleep.

### Strengths and limitations

This study has several strengths, one being the use of a rigorous translation method that ensures both accuracy and cultural relevance. Thorough psychometric evaluations were also conducted, which included assessments of internal consistency, test-retest reliability, convergent validity, and CFA. The results of these methods offered sufficient preliminary support for the reliability and validity of the Arabic JSS.

Some limitations should be noted, however. First, the sample was relatively small, and participants were recruited through convenience sampling via social media platforms. This resulted in selection bias, thus reducing the generalizability of the results. Indeed, females (84%) and adults under 35 years old (94%) were overrepresented in the sample, which could further minimize generalizability, as age and sex differences have consistently been found in research on sleep [67]. While no significant differences were found between these groups in the present study, it could be that the scale was too brief to detect such differences. Future research should examine measurement properties in larger, randomly selected samples. Furthermore, the scale’s primary intended use as a screening measure for sleep disturbances is limited, as it has yet to be tested on a clinical sample. Future research should also address this issue. Finally, the use of self-report measures could have made participants vulnerable to response bias, possibly affecting the validity of the scale.

Self-report measures such as the JSS rely on subjects’ own perceptions, which can introduce response bias such as exaggeration or selective memory [68]. Future studies could include objective sleep assessments such as actigraphy to complement self-reports. Actigraphy uses motion sensors to estimate sleep parameters, providing more objective data on sleep patterns. However, self-reports capture subjective sleep quality perceptions that objective tools miss. A combination of self-reports and objective measures may provide the most accurate, comprehensive understanding of sleep. Further research on the relationships between subjective and objective sleep data could elucidate the role of response bias in self-reports.

## Conclusions

This study translated the JSS into Arabic and demonstrated that the Arabic version has good initial psychometric properties. These findings can lead researchers and clinicians to use a reliable and valid tool for assessing self-reported sleep quality and disturbances in Arabic-speaking populations. Further validation research is warranted, particularly using randomized sampling methods and testing within clinical populations. Overall, the Arabic JSS can aid in obtaining a greater understanding of sleep health among Arabic speakers and facilitate the provision of culturally appropriate interventions when sleep difficulties arise.

### Electronic supplementary material

Below is the link to the electronic supplementary material.

Supplementary Material 1

## Data Availability

The data that support the findings of this study are available from the corresponding author upon request.
