# Supplementary material for: Translating and establishing the psychometric properties of the Jenkins Sleep Scale for Arabic-speaking individuals
Source: BMC Psychiatry. 2024 Mar 28;24:236. doi: 10.1186/s12888-024-05714-2 (PMC10976800; doi:10.1186/s12888-024-05714-2)
Supplement: Supplementary file 1 — Supplementary Material 1 [file 12888_2024_5714_MOESM1_ESM.docx]

**Supplementary material 1: Measurement invariance across age groups, sexes, and marital status**

| **Measurement invariance across age groups (less than 35 years vs 35+ years)** | | | | | |  |  |  |  |
| --- | --- | --- | --- | --- | --- | --- | --- | --- | --- |
| Model | CFI | RMSEA | SRMR | TLI | Model Comparison | ΔCFI | ΔRMSEA | ΔSRMR | ΔTLI |
| Configural | 0.98 | 0.1 | 0.03 | 0.93 | Base | - | - | - | - |
| Metric | 0.98 | 0.06 | 0.03 | 0.93 | Configural versus metric | 0 | 0.04 | 0 | 0 |
| Scalar | 0.98 | 0.06 | 0.03 | 0.93 | Metric versus scalar | 0 | 0 | 0 | 0 |
| Strict | 0.98 | 0.05 | 0.03 | 0.93 | Scalar versus strict | 0 | 0.01 | 0 | 0 |
|  |  |  |  |  |  |  |  |  |  |
| **Measurement invariance across sexes (male vs female)** | | | | | |  |  |  |  |
| Model | CFI | RMSEA | SRMR | TLI | Model Comparison | ΔCFI | ΔRMSEA | ΔSRMR | ΔTLI |
| Configural | 0.99 | 0.07 | 0.02 | 0.97 | Base | - | - | - | - |
| Metric | 0.98 | 0.07 | 0.03 | 0.97 | Configural versus metric | 0.01 | 0 | 0.01 | 0 |
| Scalar | 0.98 | 0.05 | 0.04 | 0.98 | Metric versus scalar | 0 | 0.02 | 0.01 | 0.01 |
| Strict | 0.98 | 0.04 | 0.04 | 0.99 | Scalar versus strict | 0 | 0.01 | 0 | 0.01 |
|  |  |  |  |  |  |  |  |  |  |
| **Measurement invariance across marital status (single vs married)** | | | | | |  |  |  |  |
| Model | CFI | RMSEA | SRMR | TLI | Model Comparison | ΔCFI | ΔRMSEA | ΔSRMR | ΔTLI |
| Configural | 0.97 | 0.11 | 0.03 | 0.93 | Base | - | - | - | - |
| Metric | 0.96 | 0.1 | 0.04 | 0.92 | Configural versus metric | 0.01 | 0.01 | 0.01 | 0.01 |
| Scalar | 0.96 | 0.09 | 0.05 | 0.95 | Metric versus scalar | 0 | 0.01 | 0.01 | 0.03 |
| Strict | 0.95 | 0.09 | 0.04 | 0.94 | Scalar versus strict | 0.01 | 0 | 0.01 | 0.01 |
